# Minority race and male sex as risk factors for non-beneficial gastrostomy tube placements after stroke

**DOI:** 10.1371/journal.pone.0191293

**Published:** 2018-01-19

**Authors:** Roland Faigle, Joseph A. Carrese, Lisa A. Cooper, Victor C. Urrutia, Rebecca F. Gottesman

**Affiliations:** 1 Department of Neurology, Johns Hopkins University School of Medicine, Baltimore, MD, United States of America; 2 Department of Medicine, Johns Hopkins University School of Medicine and the Johns Hopkins Berman Institute of Bioethics, Johns Hopkins University, Baltimore, MD, United States of America; 3 Department of Medicine, Johns Hopkins University School of Medicine, Baltimore, MD, United States of America; Spectrum Health, UNITED STATES

## Abstract

**Background:**

Percutaneous endoscopic gastrostomy (PEG) tubes are widely used for enteral feeding after stroke; however, PEG tubes placed in patients in whom death is imminent are considered non-beneficial.

**Aim:**

We sought to determine whether placement of non-beneficial PEG tubes differs by race and sex.

**Design and setting/participants:**

In this retrospective cohort study, inpatient admissions for stroke patients who underwent palliative/withdrawal of care, were discharged to hospice, or died during the hospitalization, were identified from the Nationwide Inpatient Sample between 2007 and 2011. Logistic regression was used to evaluate the association between race and sex with PEG placement.

**Results:**

Of 36,109 stroke admissions who underwent palliative/withdrawal of care, were discharge to hospice, or experienced in-hospital death, a PEG was placed in 2,258 (6.3%). Among PEG recipients 41.1% were of a race other than white, while only 22.0% of patients without PEG were of a minority race (p<0.001). The proportion of men was higher among those with compared to without a PEG tube (50.0% vs. 39.2%, p<0.001). Minority race was associated with PEG placement compared to whites (OR 1.75, 95% CI 1.57–1.96), and men had 1.27 times higher odds of PEG compared to women (95% CI 1.16–1.40). Racial differences were most pronounced among women: ethnic/racial minority women had over 2-fold higher odds of a PEG compared to their white counterparts (OR 2.09, 95% CI 1.81–2.41), while male ethnic/racial minority patients had 1.44 increased odds of a PEG when compared to white men (95% CI 1.24–1.67, p-value for interaction <0.001).

**Conclusion:**

Minority race and male sex are risk factors for non-beneficial PEG tube placements after stroke.

## Introduction

Stroke is a leading causes of disability and mortality in the United States[1, 2]. Dysphagia, or difficulty swallowing, can be identified in up to 50% of acute stroke patients[3, 4], and in some patients persists beyond the acute care hospitalization[3, 5]. While nasogastric (NG) tubes may be used for enteral feeding for up to several weeks, percutaneous endoscopic gastrostomy (PEG) tubes are commonly placed in stroke patients when dysphagia is expected to persist beyond 4 to 6 weeks[6–9].

Procedures and interventions intended to benefit patients during their recovery process after the acute care hospitalization, such as PEG tubes, are considered non-beneficial in patients who die during their acute care hospitalization or in whom death is imminent[8, 10]. Stroke patients are particularly susceptible to receive such non-beneficial interventions because of the associated high rates of mortality, and variable use and timing of withdrawal of life-sustaining treatments in this patient population[11–13]. Risk factors for withdrawal of treatment and do not resuscitate (DNR) orders in stroke patients have previously been investigated[11, 12, 14]. African Americans are less likely to utilize withdrawal of treatment and DNR orders compared to their white counterparts. Similarly, women are more likely to undergo withdrawal of treatment compared to men[11, 12, 14].

The impact of withdrawal of treatment orders and in-hospital mortality on non-beneficial procedures in stroke patients has not been studied systematically. Patients in whom death is imminent may unnecessarily be subjected to procedures typically reserved for those who are expected to survive beyond the acute care hospitalization. Such interventions, including PEG tubes, may be appropriate only if the functional status at the time of the procedure is sustainable and deemed acceptable by the patient and/or the patient’s decision-maker(s). Since clinical course and outcomes after stroke are frequently uncertain during the first few days after hospital admission, placement of a PEG tube early on during the hospital course may be premature in patients with uncertain prognosis[10]. Since NG tubes may serve as a viable alternative to PEG tubes for up to several weeks, PEG tubes are typically placed during the later course of the hospitalization, after patients and their decision-makers have had ample time to deliberate prognosis and goals of care[8]. Because PEG placement constitutes a surgical procedure associated with additional risks, costs, and no clear medical benefit over NG tubes during the first few weeks, PEG tubes are discouraged unless it is anticipated that they will be utilized beyond the acute hospitalization[8, 10, 15]. Therefore, PEG tubes placed in patients who subsequently die while in the hospital, undergo palliative care or care withdrawal, and those who are discharged to hospice are considered non-beneficial[10]. In the present study we aimed to determine the role of race and sex as risk factors for non-beneficial PEG placements in stroke patients, as defined by PEG placement in patients who underwent palliative/withdrawal of care, were discharged to hospice, or failed to survive their hospitalization.

## Methods

### Data source

Data were obtained from the Nationwide Inpatient Sample (NIS), part of the Healthcare Cost and Utilization Project (HCUP)[16]. The NIS is the largest all-payer inpatient database in the US, representing a 20% stratified sample of all admissions to non-federal US hospitals. All diagnoses and procedures are recorded using *International Classification of Diseases* version 9 Clinical Modification (ICD9-CM) codes. Detailed information on the design and contents of the NIS is available at http://www.hcup-us.ahrq.gov. Because NIS data are publically available and contain no personal identifying information, this study was exempt from institutional review board approval.

### Case selection

We identified adult cases with primary diagnosis of non-traumatic ICH and ischemic stroke by using ICD9-CM codes 431, 433.01, 433.11, 433.21, 433.31, 433.81, 433.91, 434.01, 434.11, 434.91, and 436 between 2007 and 2011[17]. Because the unit of observation in NIS is discharge after hospitalization, cases transferred to another hospital were excluded in order to prevent double counting of the same patient. This algorithm has been shown to identify acute ischemic and hemorrhagic stroke with high sensitivity and specificity[18, 19]. Cases with missing information on race/ethnicity and sex, the primary exposures of interest, were excluded.

### Definition of a non-beneficial PEG

We restricted the study population to cases that survived the first three days of their hospitalization, but subsequently died while in the hospital, underwent palliative/withdrawal of care, or were discharged to hospice; we considered placement of a PEG tube in this population as non-beneficial. Use of palliative/withdrawal of care was identified by ICD9-CM code V66.7. This code identifies documented use of palliative care measures irrespective of the delivery mode (i.e. via a palliative care consultation service or integrated into routine clinical practice by the care team), and is coded when terms such as comfort care, end-of-life care, and hospice care (or similar terms), are written in the patient record[20].

Since there is no previously uniformly accepted definition of a non-beneficial PEG, we performed sensitivity analysis by analyzing PEG placement only among patients who died, underwent withdrawal of care, or were discharged to hospice within 14 days of admission.

### Primary exposures and outcome of interest

The primary exposures of interest were race/ethnicity and sex. The primary outcome of interest was placement of a non-beneficial PEG as identified by ICD9-CM procedure code 43.11.

### Comorbidity and severity adjustment

We calculated the Charlson comorbidity index, a weighted score of 17 different comorbidities validated for outcome adjustment for analyses of administrative data sets using ICD9-CM codes[21, 22], for each patient. Case severity was determined using the all patient refined diagnosis-related groups (APR-DRGs)[23]. The APR-DRG algorithm is a validated and reliable indicator of mortality, and is commonly used as a severity indicator in studies relating to stroke[24].

### Statistical analysis

Comparisons of sociodemographic, hospital-level, and clinical characteristics among patients with and without PEG tube were made using Chi^2^ and Wilcoxon rank-sum tests for categorical and continuous variables, respectively. Univariable logistic regression was performed to determine the unadjusted association of PEG tube placement with race and sex, respectively. Multivariable models were adjusted for age, hospital characteristics (teaching status, bed size, location, region, and annual volume of stroke cases), discharge quarter, weekend admission, modified Charlson Comorbidity Index, APR-DRG severity subclass, insurance status, median household income per patient’s ZIP code, hypertension, diabetes mellitus, dyslipidemia, coronary artery disease, peripheral vascular disease, congestive heart failure, atrial fibrillation, valvular disease, anemia, thrombocytopenia, alcohol abuse, drug abuse, chronic kidney disease, and medical complications, such as pneumonia, urinary tract infection, sepsis, gastrointestinal bleeding, deep vein thrombosis, and pulmonary embolism. For the primary analysis, we excluded observations with missing information on the primary exposures of interest; since the variable race had substantial missingness (15.1%), we performed sensitivity analysis including imputed values for race via multiple imputation via chained equations (MICE) in order to reduce potential bias as a result of missing data. We used a Generalized Estimation Equations (GEE) approach to account for clustering of patients within hospitals. Statistical analysis was performed using STATA version 13 (*Stata Statistical Software*: *Release 13*. College Station, TX). A p-value of <0.05 was considered statistically significant. 95% confidence intervals are reported. Statistical interaction between race and sex on PEG placement was explored.

## Results

### Patient characteristics

Among the 36,109 cases that met all inclusion criteria (S1 Fig), 2,258 (6.3%) underwent PEG placement, thus termed non-beneficial PEG placements. Patients who received a PEG tube were more likely to be male (50.0% vs. 39.2%, p<0.001) than were patients who did not receive a PEG. While 7.8% of men received a non-beneficial PEG, the percentage of women receiving a non-beneficial PEG tube was 5.2% (p<0.001). While only 22.0% of patients without PEG were of a minority race, 41.1% of all PEG recipients were of a race other than white (p<0.001). Among black patients 12.5% received a PEG, compared to 4.8% receiving a PEG among white patients (p<0.001). Among all ethnic/racial minorities, 11.1% received a PEG. Further baseline characteristics are presented in Table 1.

**Table 1:** Baseline characteristics of the study population comprised of stroke admissions with eventual palliative/withdrawal of care, discharge to hospice, or in-hospital death, stratified by PEG status (n = 36,109). APR-DRG: all patient refined diagnosis-related group; PEG: percutaneous endoscopic gastrostomy. P-values compare patients with and without PEG.

Characteristics	No PEG *(n = 33*,*851)*	PEG *(n = 2*,*258)*	p-value
**Age–**years: median (IQR)	82 (72–88)	77 (65–85)	<0.001
**Male–**n (%)	13,262 (39.2)	1,129 (50.0)	<0.001
**Race**–n (%)			<0.001
White	26,406 (78.0)	1,331 (59.0)	
Black	3,570 (10.6)	512 (22.7)	
Hispanic	1,969 (5.8)	230 (10.2)	
Asian or Pacific Islander	896 (2.7)	75 (3.3)	
Other	1,010 (3.0)	110 (4.9)	
**Stroke subtype**			0.001
Ischemic	24,299 (71.8)	1,688 (74.8)	
Intracerebral Hemorrhage	9,552 (28.2)	570 (25.2)	
**Primary expected payer**–n (%)			<0.001
Private Insurance	4,171 (12.3)	275 (12.2)	
Medicare	26,246 (77.5)	1,592 (70.5)	
Medicaid	1,465 (4.3)	240 (10.6)	
Self-pay	980 (2.9)	88 (3.9)	
No charge	907 (2.7)	58 (2.6)	
Missing	82 (0.2)	<10 (0.2)	
**Median household income for patient's ZIP code: quartiles–n (%)**			<0.001
Quartile 1	8,682 (25.7)	751 (33.3)	
Quartile 2	8,478 (25.1)	552 (24.5)	
Quartile 3	8,138 (24.0)	492 (21.8)	
Quartile 4	7,880 (23.3)	402 (17.8)	
Missing	673 (2.0)	61 (2.7)	
**Hospital geographic region**–n (%)			0.001
Northeast	7,044 (20.8)	471 (20.9)	
Midwest	5,391 (15.9)	293 (13.0)	
South	14,254 (42.1)	1,103 (48.9)	
West	7,162 (21.2)	391 (17.3)	
**Hospital location**–n (%)			<0.001
Rural	3,293 (9.7)	155 (6.9)	
Urban	30,157 (89.1)	2,071 (92.7)	
Missing	401 (1.2)	32 (1.4)	
**Teaching Hospital**–n (%)	17,307 (51.1)	1,102 (48.8)	0.077
**Hospital bed size**–n (%)			<0.001
Small/Medium	11,031 (32.6)	606 (26.8)	
Large	22,419 (66.2)	1,620 (71.7)	
Missing	401 (1.2)	32 (1.4)	
**Charlson comorbidity index**			<0.001
1	7,065 (20.9)	375 (16.6)	
2	5,455 (16.1)	334 (14.8)	
3	8,033 (23.7)	541 (24.0)	
≥4	13,298 (39.3)	1,008 (44.6)	
**Hypertension**–n (%)	25,600 (75.6)	1,684 (74.6)	0.263
**Diabetes Mellitus**–n (%)	9,097 (26.9)	729 (32.3)	<0.001
**Dyslipidemia**–n (%)	9,918 (29.3)	625 (27.7)	0.101
**Coronary artery disease**–n (%)	5,558 (16.4)	328 (14.5)	0.018
**Congestive heart failure**–n (%)	7,674 (22.7)	615 (27.2)	<0.001
**Atrial fibrillation**–n (%)	12,963 (38.3)	808 (35.8)	0.017
**Chronic kidney disease**–n (%)	5,312 (15.7)	463 (20.5)	<0.001
**Anemia**–n (%)	5,146 (15.2)	550 (24.4)	<0.001
**Thrombocytopenia**–n (%)	1,222 (3.6)	119 (5.3)	<0.001
**Alcohol abuse**–n (%)	1,041 (3.1)	108 (4.8)	<0.001
**Drug abuse**–n (%)	424 (1.3)	51 (2.3)	<0.001
**APR-DRG: loss of function**			<0.001
Minor/Moderate	6,951 (20.5)	53 (2.4)	
Major	14,632 (43.2)	569 (25.2)	
Extreme	12,266 (36.2)	1,636 (72.5)	
Missing	<10 (0.0)	0 (0.0)	

### Minority race and male sex are associated with increased risk of non-beneficial PEG

In univariable analysis, the odds of PEG placement were significantly higher for patients of a minority race compared to whites: OR 2.85, 95% CI 2.55–3.17 in blacks, OR 2.32, 95% CI 2.00–2.69 in Hispanics, and OR 1.66, 95% CI 1.30–2.12 in Asians/Pacific Islanders. These results persisted after multivariable adjustment for age, comorbidities, medical complications including dysphagia, and hospital characteristics (Table 2). Taken together, minority patients had 1.75 times higher odds of PEG compared to whites in the adjusted model (95% CI 1.57–1.96). Male sex was associated with PEG placement in univariable analysis (OR 1.55, 95% CI 1.43–1.69), and this association persisted in adjusted models (OR 1.27, 95% CI 1.16–1.40; Table 2). To reduce the potential for bias from missing data, we performed sensitivity analysis after multiple imputation of the missing race values. This sensitivity analysis yielded similar results as complete-case analysis with regard to effect size and statistical significance (S1 Table).

**Table 2:** Race and sex as determinants of non-beneficial PEG tubes after stroke.

	Crude			Adjusted*		
Variable	OR	95% CI	p-value	OR	95% CI	p-value
**Race**						
White	1.00 (ref)			1.00 (ref)		
Black	2.85	2.55–3.17	<0.001	1.85	1.62–2.12	<0.001
Hispanic	2.32	2.00–2.69	<0.001	1.67	1.42–1.97	<0.001
Asian/Pacific Islander	1.66	1.30–2.12	<0.001	1.46	1.08–1.98	0.014
Other	2.16	1.76–2.65	<0.001	1.76	1.34–2.30	<0.001
**All Minorities**	2.47	2.26–2.70	<0.001	1.75	1.57–1.96	<0.001
**Sex**						
Female	1.00 (ref)			1.00 (ref)		
Male	1.55	1.43–1.69	<0.001	1.27	1.16–1.40	<0.001

*Model adjusted for age, hospital teaching status, hospital bed size, hospital location, hospital region, and annual volume of stroke cases, discharge quarter, weekend admission status, modified Charlson Comorbidity Index, APR-DRG severity subclass, insurance status, median household income per patient’s ZIP code, hypertension, diabetes mellitus, dyslipidemia, coronary artery disease, peripheral vascular disease, congestive heart failure, atrial fibrillation, valvular disease, anemia, thrombocytopenia, alcohol abuse, drug abuse, chronic kidney disease, pneumonia, urinary tract infection, sepsis, gastrointestinal bleeding, deep vein thrombosis, and pulmonary embolism.

### Racial differences in non-beneficial PEG tubes after stroke vary by sex

**Fig 1:**
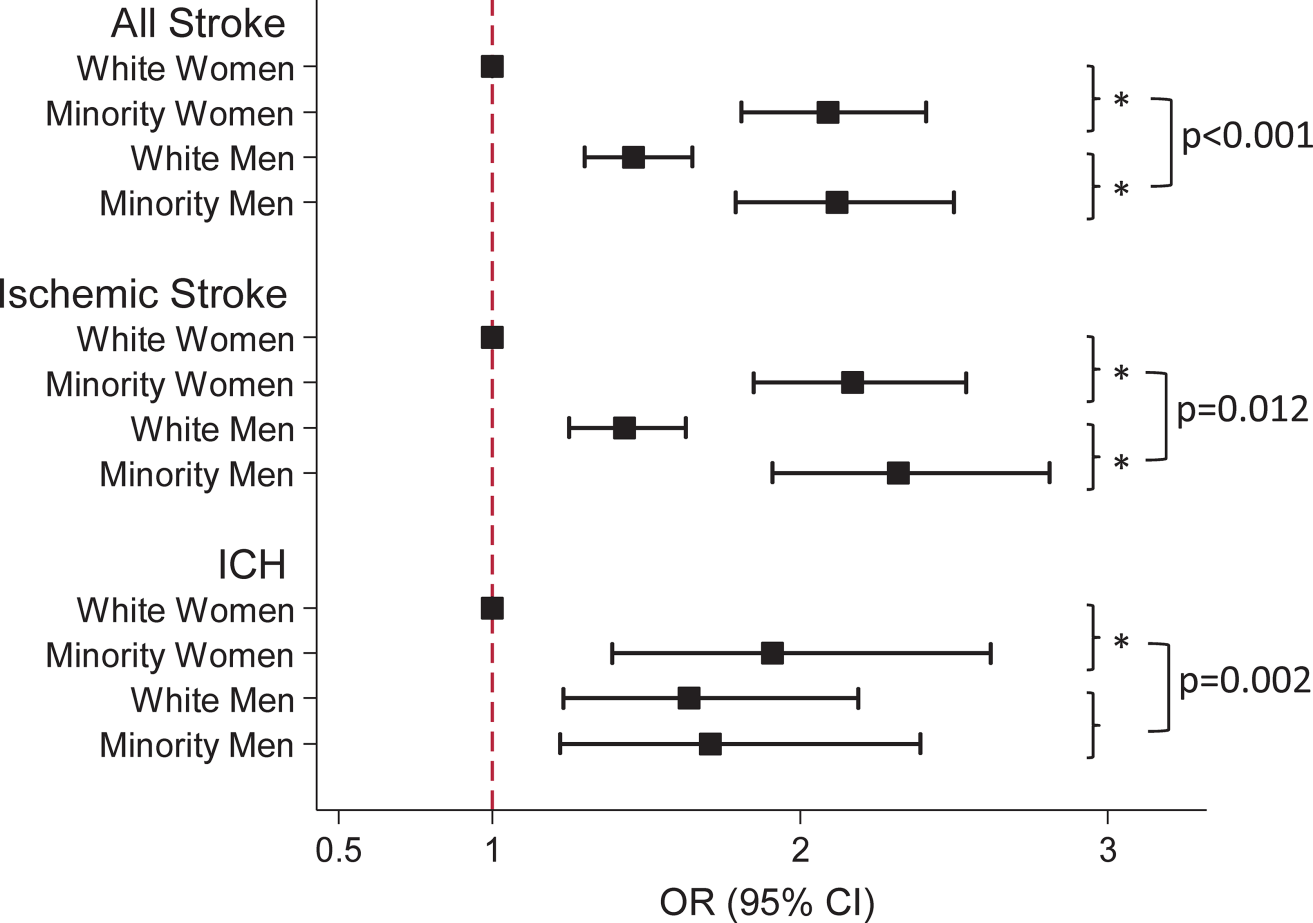
Graphic representation of odds ratios of PEG among ethnic/racial minority women, white men, and ethnic/racial minority men, compared to white women as reference. Data are presented for all stroke (top panel), and stratified by stroke subtype (medium and lower panel), respectively. * indicates a p-value <0.001 for comparison of PEG in ethnic/racial minority vs. white for each sex. P-values for interaction following square brackets compare the odds of PEG in ethnic/racial minorities vs. white between men and women. OR: odds ratio; CI: confidence interval; ICH: intracerebral hemorrhage.

In the entire study population comprised of patients with in-hospital mortality, discharge to hospice, or undergoing palliative/withdrawal of care, non-beneficial PEG tubes after any stroke were most common among ethnic/racial minority men (11.9%), while only 3.8% of white women received a PEG tube (Table 3). Similarly, the adjusted odds of PEG were highest among ethnic/racial minority men (OR 2.10, 95% CI 1.78–2.48, compared to white women; Table 3). Differences by race were most pronounced among women: ethnic/racial minority women had over 2-fold higher odds of PEG compared to their white counterparts (OR 2.08, 95% CI 1.81–2.41), while male ethnic/racial minority patients had 1.44-fold increased odds of a PEG when compared to white men (95% CI 1.24–1.67, p-value for interaction <0.001; Fig 1). The odds of non-beneficial PEG tubes did not differ between ethnic/racial minority women and men (OR 1.01, 95% CI 0.86–1.18 in ethnic/racial minority men compared to minority women).

**Table 3:** Odds of non-beneficial PEG tubes among the different race/sex groups stratified by stroke type.

Variable	% PEG	Crude OR (95% CI) of PEG	Adjusted* OR (95% CI) of PEG
**All Stroke**			
White Female	3.8	1 (ref)	1 (ref)
Minority Female	10.4	2.98 (2.63–3.37)	2.08 (1.81–2.41)
White Male	6.5	1.76 (1.58–1.97)	1.46 (1.30–1.64)
Minority Male	11.9	3.45 (3.03–3.91)	2.10 (1.78–2.48)
**Ischemic Stroke**			
White Female	4.1	1 (ref)	1 (ref)
Minority Female	11.1	2.96 (2.57–3.41)	2.17 (1.85–2.54)
White Male	6.8	1.73 (1.53–1.96)	1.43 (1.25–1.63)
Minority Male	13.0	3.54 (3.04–4.11)	2.32 (1.91–2.81)
**Intracerebral hemorrhage**			
White Female	2.9	1 (ref)	1 (ref)
Minority Female	9.0	3.35 (2.59–4.34)	1.91 (1.39–2.62)
White Male	5.6	2.02 (1.59–2.57)	1.64 (1.23–2.19)
Minority Male	10.1	3.85 (2.99–4.95)	1.71 (1.22–2.39)

*Model adjusted for age, hospital teaching status, hospital bed size, hospital location, hospital region, and annual volume of stroke cases, discharge quarter, weekend admission status, modified Charlson Comorbidity Index, APR-DRG severity subclass, insurance status, median household income per patient’s ZIP code, hypertension, diabetes mellitus, dyslipidemia, coronary artery disease, peripheral vascular disease, congestive heart failure, atrial fibrillation, valvular disease, anemia, thrombocytopenia, alcohol abuse, drug abuse, chronic kidney disease, pneumonia, urinary tract infection, sepsis, gastrointestinal bleeding, deep vein thrombosis, and pulmonary embolism.

Among patients with ischemic stroke, ethnic/racial minority men and women both had similarly increased odds of non-beneficial PEG when compared to white women (OR 2.32, 95% CI 1.91–2.81, for ethnic/racial minority men vs. OR 2.17, 95% CI 1.85–2.54, for ethnic/racial minority women; Table 3). Increased odds of PEG for ethnic/racial minorities vs. whites were observed among both men and women after ischemic stroke, however, were more pronounced among women (p-value for interaction 0.012; Fig 1). In patients with ICH, the odds of PEG was significantly increased in ethnic/racial minority women (OR 1.91, 95% CI 1.39–2.62), white men (OR 1.64, 95% CI 1.23–2.19), and ethnic/racial minority men (OR 1.71, 95% CI 1.22–2.39), when compared to white women (Table 3). Racial differences for PEG placement after ICH were seen among women, but not men (p-value for interaction 0.002; Fig 1).

### Sensitivity analysis: Patients with death or discharge to hospice within 14 days of admission

There is no unified consensus as to what constitutes a non-beneficial PEG tube (i.e. a PEG tube placed in a patient on hospital day 15 who weeks later succumbs to an unforeseen complication may not uniformly be regarded as “non-beneficial”). Therefore, we performed a sensitivity analysis by investigating PEG placement only among stroke patients who were discharged to hospice, died, or received end-of-life/palliative care within the first 14 days of their hospitalization. Any PEG in this population would have been placed very close to the time of death and/or relatively early in the hospital course.

Of all 2,258 PEG tubes in our original study population, 1,079 (47.8%) were placed in patients who were discharged to hospice or died within 14 days of admission. The adjusted odds of PEG in this patient population was higher in white men (OR 1.43, 95% CI 1.23–1.67), ethnic/racial minority women (OR 2.00, 95% CI 1.64–2.45), and ethnic/racial minority men (OR 2.17, 95% CI 1.74–2.72), when compared to white women (S2 Table).

## Discussion

In the present study we have identified minority race and male sex as risk factors for non-beneficial PEG placement in stroke patients. Non-beneficial procedures or interventions in patients who later transition to palliative care and treatment withdrawal and/or die during their hospitalization may in part be due to continuation of aggressive therapy despite no anticipated benefit (i.e. because of family wishes, or providers’ reluctance to ‘give up’), or absence of timely prediction of a poor outcome. Since timing and accuracy of outcome prediction should not differ by race or sex, it is more likely that unnecessary continuation of aggressive therapy among ethnic/racial minorities and men accounts for the increased rates of non-beneficial PEG tubes placed in these populations. Therefore, the higher rates of PEG tubes placed in men and ethnic/racial minority patients in our study suggests that ongoing aggressive care in some patients may result in non-beneficial procedures.

Racial differences in utilization of interventions at the end of life have been described, i.e. black patients are less likely to institute DNR orders or request withdrawal of active treatment compared to their white counterparts[14, 25]. Similarly, white patients more commonly have advanced directives[26, 27]. Prolonged aggressive care despite a low likelihood of benefit may result in higher rates of unnecessary procedures in ethnic/racial minorities. Among recipients of non-beneficial PEG tubes, ethnic/racial minorities had longer length of stay and longer time-to-PEG placement. This indicates that prolonged aggressive care may increase the risk of non-beneficial procedures by providing additional time during which non-beneficial treatment is administered.

The higher odds of non-beneficial PEG in men compared to women, as observed in our study, may reflect that women overall receive less aggressive care after stroke compared to their male counterparts[28–30]. In addition, our finding is in line with other studies suggesting that women in general are less likely to receive life-sustaining interventions and are more likely to have DNR orders[31–33]. We observed sex disparities only among white but not among ethnic/racial minority patients, although both ethnic/racial minority men and women had higher odds of PEG than white men or women. Although the reasons for this finding are not entirely clear, it may suggest a hierarchical order of disparities, i.e. racial differences in PEG placement after stroke may “trump” differences by sex, and sex disparities may only be relevant among whites.

We restricted our study population to patients in whom a PEG tube is considered non-beneficial; 6.3% of those patients received a PEG, exceeding the rate of PEG tubes placed in our total ischemic and hemorrhagic stroke population (5.1%). While some in-hospital deaths after PEG may have been unexpected and therefore difficult to predict, timely identification of patients in whom further procedures are unlikely to be beneficial may aid in reducing non-beneficial PEG placements. Although NG tubes are safe and sufficient to provide enteral nutrition for up to about 2 weeks after stroke, more than half of the patients in the present study, black and white alike, received their PEG within the first 10 days of hospitalization. This is consistent with other recent reports[34, 35]. This suggests that PEG tubes were placed prematurely in a substantial number of patients, many of which likely had no meaningful chance of recovery at the time of PEG placement as indicated by the median time from PEG to death/hospice discharge of just 6 days. Formal and objective criteria for PEG placement after stroke may help avoid PEG tubes in patients who are unlikely to benefit from it.

Our study has several limitations. The NIS does not contain clinical and physiological data on stroke volume or location, level of consciousness, or associated laboratory parameters, which may confound the described associations of race/sex with PEG placement. We attempted to mitigate this shortcoming by adjusting all regression models for the Charlson Comorbiditity Index, a validated measure of patient comorbidities in stroke[21, 36]. Miscoded and missing data may occur in large administrative datasets; however, it is unlikely that there is differential miscoding by race or sex. To address missingness for race, we performed a sensitivity analysis using multiple imputation for the race variable. Although ICD9-CM code V66.7 identifies palliative/withdrawal of care services use with high sensitivity and specificity[37], there is a possibility of under-reporting or under-coding patients who actually received palliative care or undergo care withdrawal. While we attempted a meaningful definition of a “non-beneficial PEG”, our definition is subjective, yet not exhaustive; i.e. PEG tubes placed in patients who regain swallow function and do not use their PEG after discharge may also be considered non-beneficial, but were not examined in our study.

Despite these limitations, our data suggest that PEG tubes placed in stroke patients with subsequent short-term mortality are relatively common, and that ethnic/racial minorities and men are at particular risk. In the context of engaging all patients and their next of kin decision-makers in conversations about goals of care and the appropriate use of available interventions, we propose that health care professionals carefully examine their approaches to care of ethnic/racial minorities and men when counseling families on neurological outcome and need for PEG tubes after stroke. Standardized interdisciplinary approaches for evaluation and timing of PEG placement in light of the underlying stroke prognosis may aid in minimizing placement of non-beneficial PEG tubes.

## Supporting information

S1 Fig Flow diagram indicating selection of the study population. *Not mutually exclusive.
(TIF)

S1 Table Multivariable analysis for race and sex determinants of non-beneficial PEG placement after stroke. Sensitivity analysis including imputed data for race; n = 42,235.
(DOCX)

S2 Table Sensitivity analysis: Odds of PEG among stroke patients who died or were discharge to hospice within 14 days of admission (n = 32,560). (DOCX)
