# Supplementary material for: Minority race and male sex as risk factors for non-beneficial gastrostomy tube placements after stroke
Source: PLoS One. 2018 Jan 19;13(1):e0191293. doi: 10.1371/journal.pone.0191293 (PMC5774766; doi:10.1371/journal.pone.0191293)
Supplement: S1 Table — Sensitivity analysis including imputed data for race; n = 42,235. (DOCX) [file pone.0191293.s002.docx]

S1 Table. Multivariable analysis for race and sex determinants of non-beneficial PEG placement after stroke; sensitivity analysis including imputed data for race; n=42,235.

|  | **Unadjusted** | | | **Adjusted*** | | |
| --- | --- | --- | --- | --- | --- | --- |
| **Variable** | **OR** | **95% CI** | **p-value** | **OR** | **95% CI** | **p-value** |
| **Race** |  |  |  |  |  |  |
| White | 1.00 (ref) |  |  | 1.00 (ref) |  |  |
| Black | 2.80 | 2.50-3.12 | <0.001 | 1.90 | 1.67-2.17 | <0.001 |
| Hispanic | 2.36 | 2.04-2.74 | <0.001 | 1.75 | 1.49-2.06 | <0.001 |
| Asian/Pacific Islander | 1.65 | 1.30-2.11 | <0.001 | 1.43 | 1.07-1.91 | 0.016 |
| Other | 2.15 | 1.76-2.63 | <0.001 | 1.73 | 1.34-2.23 | <0.001 |
|  |  |  |  |  |  |  |
| **Sex** |  |  |  |  |  |  |
| Female | 1.00 (ref) |  |  | 1.00 (ref) |  |  |
| Male | 1.57 | 1.45-1.70 | <0.001 | 1.29 | 1.18-1.41 | <0.001 |

*Model adjusted for age, hospital teaching status, hospital bed size, hospital location, hospital region, and annual volume of stroke cases, discharge quarter, weekend admission status, modified Charlson Comorbidity Index, APR-DRG severity subclass, insurance status, hypertension, diabetes mellitus, dyslipidemia, coronary artery disease, peripheral vascular disease, congestive heart failure, atrial fibrillation, valvular disease, anemia, thrombocytopenia, alcohol abuse, drug abuse, chronic kidney disease, pneumonia, urinary tract infection, sepsis, gastrointestinal bleeding, deep vein thrombosis, and pulmonary embolism.
