# Supplementary material for: Minority race and male sex as risk factors for non-beneficial gastrostomy tube placements after stroke
Source: PLoS One. 2018 Jan 19;13(1):e0191293. doi: 10.1371/journal.pone.0191293 (PMC5774766; doi:10.1371/journal.pone.0191293)
Supplement: S2 Table — (DOCX) [file pone.0191293.s003.docx]

S2 Table. Sensitivity analysis: Odds of PEG among stroke patients who died or were discharge to hospice within 14 days of admission (n=32,560).

| **Variable** | **% PEG** | **Crude OR (95% CI)**  **of PEG** | **Adjusted OR (95% CI)**  **of PEG** |
| --- | --- | --- | --- |
| White Female | 2.4 | 1 (ref) | 1 (ref) |
| Minority Female | 5.2 | 2.20 (1.85-2.61) | 2.00 (1.64-2.45) |
| White Male | 3.5 | 1.46 (1.26-1.69) | 1.43 (1.23-1.67) |
| Minority Male | 5.2 | 2.20 (1.81-2.66) | 2.17 (1.74-2.72) |
